# Ex vivo infection of murine precision-cut lung tissue slices with *Mycobacterium abscessus*: a model to study antimycobacterial agents

**DOI:** 10.1186/s12941-020-00399-3

**Published:** 2020-11-22

**Authors:** Carmen Amelia Molina-Torres, Oscar Noé Flores-Castillo, Irma Edith Carranza-Torres, Nancy Elena Guzmán-Delgado, Ezequiel Viveros-Valdez, Lucio Vera-Cabrera, Jorge Ocampo-Candiani, Julia Verde-Star, Jorge Castro-Garza, Pilar Carranza-Rosales

**Affiliations:** 1 Servicio de Dermatología, Hospital Universitario “José E. González”, Universidad Autónoma de Nuevo León, Monterrey, NL México; 2 Facultad de Ciencias Biológicas, Universidad Autónoma de Nuevo León, Monterrey, NL México; 3 Centro de Investigación Biomédica del Noreste, Instituto Mexicano del Seguro Social, Monterrey, NL México; 4 División de Investigación en Salud, UMAE, Hospital de Cardiología #34, Instituto Mexicano del Seguro Social, Monterrey, NL México

**Keywords:** *Mycobacterium abscess*us, Lung tissue slices, Ex vivo infection

## Abstract

**Background:**

Multidrug-resistant infections due to *Mycobacterium abscessus* often require complex and prolonged regimens for treatment. Here, we report the evaluation of a new ex vivo antimicrobial susceptibility testing model using organotypic cultures of murine precision-cut lung slices, an experimental model in which metabolic activity, and all the usual cell types of the organ are found while the tissue architecture and the interactions between the different cells are maintained.

**Methods:**

Precision cut lung slices (PCLS) were prepared from the lungs of wild type BALB/c mice using the Krumdieck^®^ tissue slicer. Lung tissue slices were ex vivo infected with the virulent *M. abscessus* strain L948. Then, we tested the antimicrobial activity of two drugs: imipenem (4, 16 and 64 μg/mL) and tigecycline (0.25, 1 and 4 μg/mL), at 12, 24 and 48 h. Afterwards, CFUs were determined plating on blood agar to measure the surviving intracellular bacteria. The viability of PCLS was assessed by Alamar Blue assay and corroborated using histopathological analysis.

**Results:**

PCLS were successfully infected with a virulent strain of *M. abscessus* as demonstrated by CFUs and detailed histopathological analysis. The time-course infection, including tissue damage, parallels in vivo findings reported in genetically modified murine models for *M. abscessus* infection. Tigecycline showed a bactericidal effect at 48 h that achieved a reduction of > 4log_10_ CFU/mL against the intracellular mycobacteria, while imipenem showed a bacteriostatic effect.

**Conclusions:**

The use of this new organotypic ex vivo model provides the opportunity to test new drugs against *M. abscessus*, decreasing the use of costly and tedious animal models.

## Background

*Mycobacterium abscessus* is an important emerging pathogen responsible for a wide spectrum of diseases, including chronic pulmonary disease, and skin and soft tissue infections. *M. abscessus* is a nontuberculous mycobacteria (NTM) found in soil and water, including municipal and household water supply systems. This species is one of the most resistant organisms to chemotherapeutic agents [1] and is therefore often referred to as the “incurable nightmare” [2]. The treatment of *M. abscessus* infections usually consists of a mixture of a macrolide plus parenteral antimicrobials, that can be either an aminoglycoside or cefoxitin, imipenem or tigecycline [3, 4]. The cure rate achieved among patients with an *M. abscessus* pulmonary infection is typically between 25 and 88% [5–7], and therapy is usually given for as long as 18–24 months, with a minimum combination of three drugs [5, 8]. In addition, these therapeutic schemes have a high cost, it has been estimated that a total of 1.4 billion dollars was spent on NTM-pulmonary disease in the USA in 2014 [9]. Therefore, there is an urgent need to develop safe and more effective drugs with anti-NTM activity. Currently, there are potential therapeutic agents in research and development for the treatment of NTM pulmonary disease, including *M. abscessus* infection. However, with the tuberculosis drug pipeline, where > 35 chemical entities are in the discovery stage, the NTM drug pipeline is nearly empty [10].

In addition, to develop more effective regimens against NTM diseases, it is necessary to implement models to help test novel drugs or compounds with potential antibiotic effects. The efficacy of drugs against *M. abscessus*, as well as other mycobacteria, is traditionally studied using in vitro and in vivo models [11–13]; however, in vitro studies cannot fully represent the complexity of the lung architecture and its impact on host–pathogen interactions, while animal models have their own limitations [14, 15]. For example, animal experiments are often poorly designed and fail to provide the proper foundation for subsequent human studies [16], and they have issues related to reproducibility and translation into preclinical studies [17].

The necessity of experimental models that provide a more accurate representation of the in vivo 3D structure of the lung than cell lines, grown as monolayers, has increased the interest in ex vivo tissue cultures [18]. With this approach, tissue explants have been successfully infected with *Mycobacterium tuberculosis*, *M. abscessus*, and *Mycobacterium avium* [19]. Similarly, we have reported a *M. tuberculosis* infection model using precision-cut lung slices (PCLS) [20]. PCLS are an ex vivo system that reflects the 3D tissue architecture, cellular composition, matrix complexity, metabolic function and immune response of the lung [21, 22]. PCLS have also been used as an infection model to study mycoplasma [23], viruses [24, 25] and bacteria [26–28]. The characteristics of this ex vivo lung model system could offer some advantages when testing compounds directed against several pathogens of the respiratory tract.

In this work, we describe the evaluation of an infection model with a virulent strain of *M. abscessus* using murine precision-cut lung tissue slices. Once infection was established, we evaluated the antimicrobial activity of tigecycline and imipenem against the infected lung slices. This model will provide valuable information for the study of *M. abscessus* pathogenesis and in the search for novel drugs against mycobacteria.

## Materials and methods

### Bacterial strains

*M. abscessus* virulent strain L948 (ATCC 19977) was grown in Middlebrook 7H9 broth and stored in vials at − 70 °C. Bacterial vials were thawed, and the colony forming units/mL (CFU/mL) were determined by serial dilution on blood agar.

### Compounds

Tigecycline and imipenem (PiSA Pharmaceutical, DF, México) were reconstituted in sterile 0.85% NaCl, and aliquots were prepared and stored at − 70 °C until further use. Krebs–Henseleit (KB) buffer (pH 7.4) was used for PCLS preparation, and DMEM/F12 supplemented with 10% bovine fetal serum, 1% insulin–transferrin–selenium and 25 mM glucose (complete medium) was used for PCLS culture.

### Minimal inhibitory concentration

Tigecycline and imipenem stock solutions were prepared at a concentration of 1 mg/mL. The MIC value for each drug against *M. abscessus* L 948 (virulent strain) was determined as recommended by the Clinical and Laboratory Standards Institute (CLSI) document M24-A2 using a broth microdilution method [29]. The final drug concentration range was 0.25 to 64 μg/mL. The MIC values were determined after 72 h of incubation at 30 °C. Quality control testing was performed using *Staphylococcus aureus* ATCC 29213.

### Precision-cut lung tissue slices preparation

The PCLS were prepared from 8 to 10-week-old male BALB/c mice (Harlan Laboratories SA de CV, México). The mice were euthanized with an overdose of sodium pentobarbital (6 mg/100 g) following institutional and international guidelines for the humanitarian care of animals used in experimental work. Afterwards, the pleural cavity was exposed under aseptic conditions, and the trachea was cannulated to infiltrate the lungs with 0.7% low-gelling temperature agarose in basal DMEM/F12 medium at 37 °C. The lungs were allowed to cool on ice to obtain a firm consistency and were then excised and immersed in sterile Krebs–Henseleit (KB) buffer (pH 7.4 at 4 °C). Cylindrical lung tissue cores of 5 mm diameter were obtained; from these, 350–400 µm thick tissue slices were prepared using a Krumdieck^®^ tissue slicer (Munford, AL, USA), with a constant flow of oxygenated KB buffer (4 °C, 95:5% O_2_:CO_2_). The lung slices were placed in 24-well microplates (one per well) with 1 mL per well of DMEM/F12 medium. The plates were pre-incubated for 4 h at 37 °C, 5% CO_2_, with a slow agitation at ~ 25 rpm, and the medium was changed four times every 30 min to remove the agarose. Afterwards, the basal viability of the lung tissue slices was determined by Alamar Blue™ assay [30]. Fluorescence (at 530 nm excitation/590 nm emission wavelengths) was determined in the FLx800 Multi-detection Microplate Reader (Biotek Instruments, Winooski, VT, USA).

### Infection of the PCLS and intracellular activity of the antibacterial drugs

After removing the agarose from the tissue, 250 μL of DMEM/F12 complete medium was added to the PCLS in the 24-well microplates and inoculated with* M. abscessus* ATCC 19977 (1.5 × 10^7^ CFU in total, per slice). One group of slices was processed immediately for histopathological analysis. The remaining slices were inoculated and incubated at 37 °C with 5% CO_2_ for 1 h without agitation. Then, 1 mL of complete DMEM/F12 was added, followed by incubation for 1 h. After removing the medium, the slices were washed twice with 500 μL of PBS buffer with amikacin (200 μg/mL) to eliminate any extracellular *M. abscessus*. The PCLS were washed again, and the antimicrobial compounds diluted in complete DMEM/F12 medium were added by triplicate as follows: imipenem at 4, 16 and 64 μg/mL and tigecycline at 0.25, 1 and 4 μg/mL, followed by incubation for 12, 24 and 48 h. The CFUs were determined by transferring the PCLS to a microcentrifuge tube containing 1 mL of distilled water and sterile glass beads and washing twice. The intracellular bacteria were released using a sterile scalpel macerating the tissue until it had disintegrated, followed by vortexing with 1 mL of PBS-Tween 20 solution for 5 min; CFUs were determined by plating on blood agar. The experiments were performed in triplicate, and the data were expressed as the log_10_. In all cases, a control group, untreated with antibiotic, was prepared for each corresponding time point.

### PCLS histopathological analysis

After incubation time the infected and control lung tissue slices were fixed in 10% neutral formalin for 24 h at room temperature and then embedded in paraffin using conventional histological techniques. Sections of 5 µm thickness were obtained from the embedded tissues using a microtome (American Optical, Buffalo NY, USA), mounted on glass slides, and stained with hematoxylin and eosin (H&E) or Ziehl–Neelsen (ZN) dyes. The Oil red-O stain kit KTORO (StatLab., McKinney TX, USA) was used to perform the Oil red-O classic lipid stain to confirm the presence of foamy macrophages. Frozen-infected slices were carefully embedded in Tissue-Tek^®^ on the mold of the cryostat. Sections of 10–12 μm-thick were prepared using a Leica CM1850 cryostat (Buffalo Grove, IL, USA); they were placed on glass slides and let them dry for 30 min at room temperature; the staining process was done according to the manufacturer instructions. Briefly: slides with the frozen sections were placed in 10% neutral formalin for 2–5 min then rinsed in tap water. Slides were subsequently placed in propylene glycol for 2 min, stained 6 min with oil red O working solution at 60 °C; placed 1 min in 85% propylene glycol, rinsed twice with distilled water; stained 1 min with modified Mayer’s hematoxylin, rinsed twice with tap water followed by 2 changes of distilled water, and mounted with aqueous glycerol-jelly medium. All the stained sections were observed using a Zeiss Axiostar Plus Brightfield Microscope (Jena, Germany); photographs were obtained with a 5.0 Moticam camera (Richmond, BC, Canada). All sections were observed using a Zeiss Axiostar Plus Brightfield Microscope (Jena, Germany); photographs were obtained with a 5.0 Moticam camera (Richmond, BC, Canada).

### Statistical analysis

For the descriptive analysis, all the values are presented as mean values and standard deviations (± SD). The data were compared by using the Student’s t-test and Bonferroni’s multiple comparison posttest, considering *P* < 0.05 as significant.

## Results

PCLS of adequate quality were obtained to perform all the experiments. Lung slices with thickness, diameter, and macroscopic integrity were selected.

### Histopathologic analysis

**Fig. 1:**
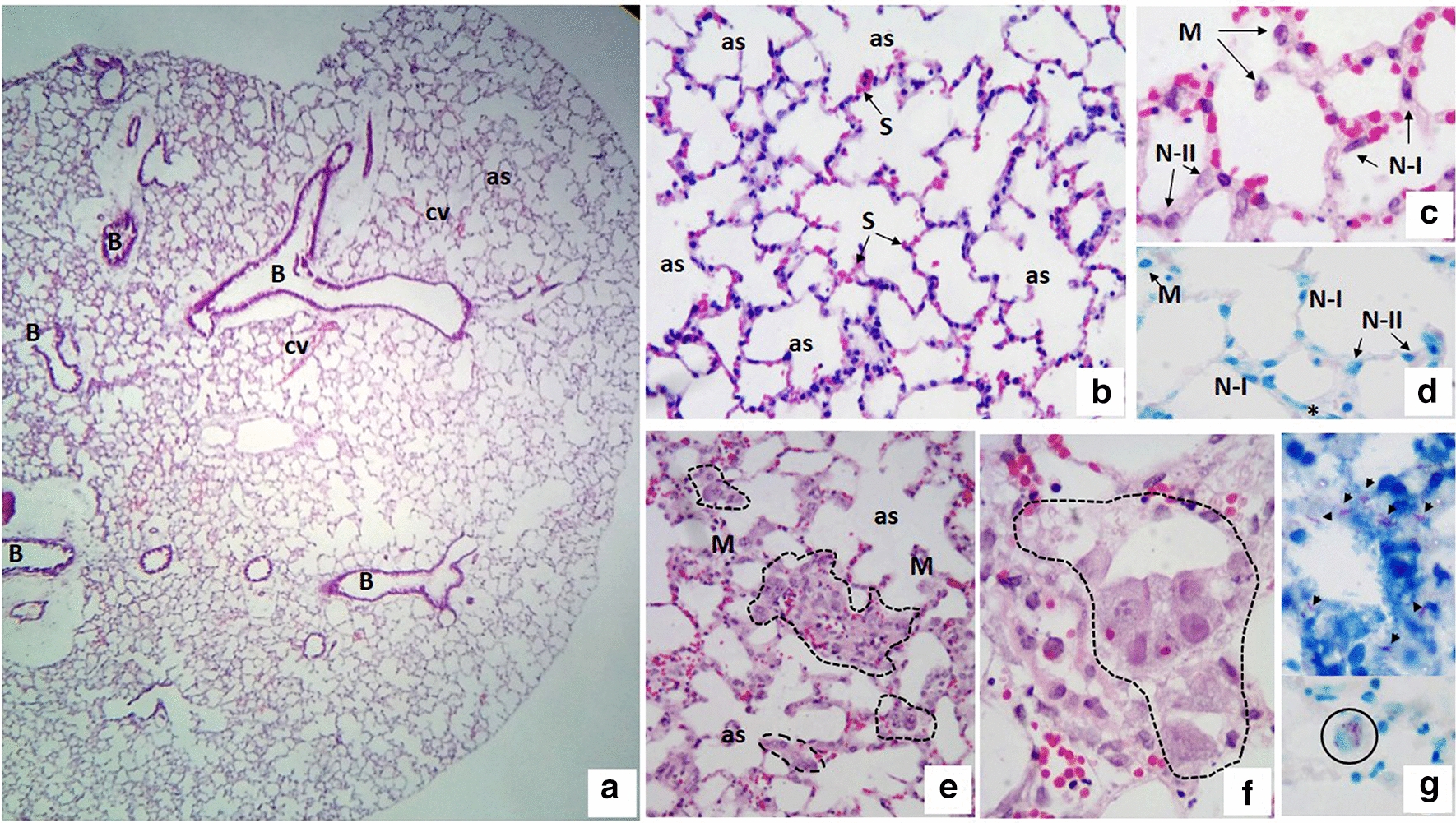
Uninfected (control) and *Mycobacterium abscessus*-infected murine precision-cut lung slices. **a**–**c** Uninfected PCLS cultured for 48 h show normal pulmonary histology, with empty alveolar spaces (as) and alveolar septa (S) constituted by fibrous connective tissue and congestive blood vessels. The walls of the septa are lined by type I pneumocytes (N-I) and type II pneumocytes (N-II). Some isolated macrophages are observed in the wall and in the lumen of the alveolus (M). **d** Uninfected PCLS (control), macrophages (M), and type I (NI) and type II (NII) pneumocytes are observed. The ZN staining is negative. **e**, **f** In PCLS infected with *M. abscessus* for 24 h, a chronic inflammatory reaction is observed, mainly composed by lymphocytes and foamy macrophages forming conglomerates (dotted area). **g** Representative photomicrograph of infected slices stained with ZN that are positive for the presence of mycobacteria; *M. abscessus* is found in the lumen of the alveoli (arrowheads) and intracellularly infecting the foamy macrophages (circle). H&E staining: (**a**–**c**, **e**, **f**), ZN staining: (**d**, **g**). Total magnification: ×5 (**a**), ×10 (**b**, **e**), ×40 (**c**, **d**, **f**, **g**)

The morphology of freshly obtained lung tissue showed no differences with the uninfected PCLS incubated for 5 days, based on the histologic structure. Representative images of the cultured PCLS show their characteristic structural elements: typical bronchi, terminal and respiratory bronchioles, alveolar ducts, alveolar sacs, alveoli and septa. In addition to the structural elements, alveolar macrophages, and type I and II pneumocytes were observed (Fig. 1a–d). Thin blood vessels were seen in the alveolar septa, with little to no evidence of inflammatory cells in the alveolar space. After 5 days of incubation, the histologic integrity of the tissue was maintained. The viability of the lung tissue slices was 97% at 48 h and there were no significant differences in the viability of the freshly obtained (basal) and control (uninfected) slices (100 ± 5% and 116 ± 20%, respectively).

In *M. abscessus*-infected PCLS for six to 24 h, we observed a mixed inflammatory reaction (Fig. 1e–g) mainly composed of abundant foamy macrophages, lymphocytes, and plasmatic cells. Mycobacteria bacilli were found in the PCLS, particularly in the alveolar lumen, in close contact with the foamy macrophages, or intracellularly infecting these cells. At 6 h post-infection, the presence of a greater number of epithelioid cells in the alveoli, a few lymphocytes in the septa, and some septa with mild edema were observed, while the histologic architecture was still conserved. After 12 and 24 h post-infection, the alveolar septa showed more edema, vascular congestion, and extravasation of erythrocytes and lymphocytes in the septa and the alveolar spaces (Fig. 1e–g).

**Fig. 2:**
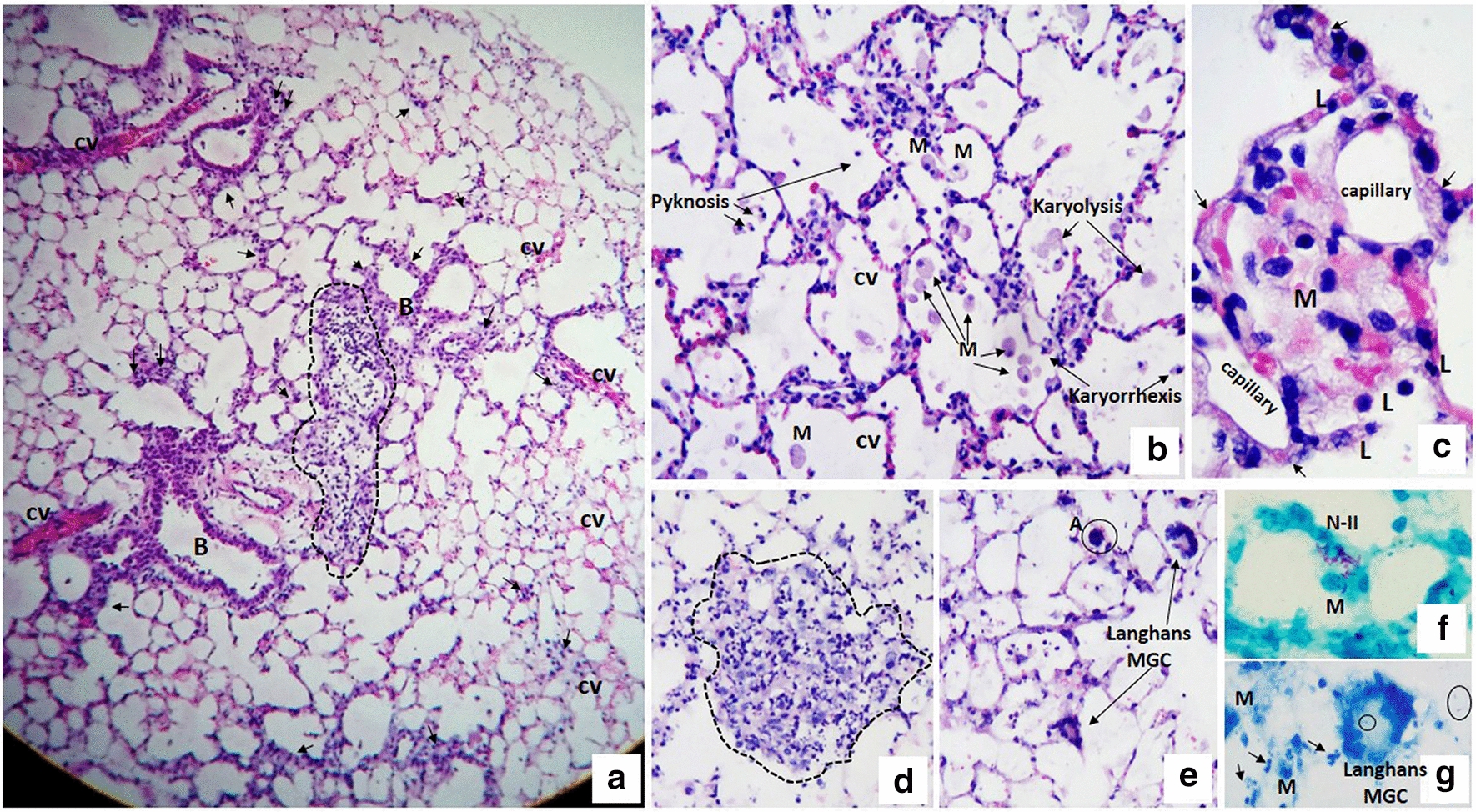
Morphological aspects of the infection of murine PCLS infected for 48 h with *M. abscessus*. **a** Panoramic view of a complete lung slice infected with *M. abscessus* for 48 h showing preserved tissue viability and an inflammatory aggregate with a loss of alveolar spaces (dotted area). Vascular congestion (vc) and discrete inflammatory infiltrate in the peribronchioles (B) with septal thickening (arrowhead) are also observed. **b** Alveolar spaces with an increase in foamy macrophages (FM), some of which show nuclear changes such as pyknosis, karyolysis and karyorrhexis. In the alveolar septa, vascular congestion and moderate inflammatory infiltrate are observed. **c** The alveolar space is occupied entirely by a conglomerate of foamy macrophages mixed with erythrocytes; the alveolar septa show discrete thickening secondary to edema, vascular congestion (arrows) and lymphocyte infiltrate (L). In addition, normal capillary vessels are observed adjacent to the septa. **d**, **e** Photomicrograph showing lung tissue with a loss of its histological structure and occupied by inflammatory infiltrates of mononuclear cells, polymorphonuclear cells, and histiocyte/macrophages (dotted area). The rest of the tissue shows septal inflammation and vascular congestion. In addition to the histological findings in the photomicrograph (**c**), multinucleated giant Langhans cells and apoptotic cells (A) are observed. **f** Abundant mycobacteria in the alveolar septum infecting type II pneumocytes (N-II) and foamy macrophages (FM). **g** Mycobacterial fragments (circles) are observed within Langhans multinucleated giant cell and extracellularly. Foamy macrophages (FM) and nuclear fragments (arrows) can also be observed. H&E staining (**a**–**e**). ZN staining (**f**, **g**). Total magnification: ×50 (**a**), ×100 (**d**, **e**), ×200 (**b**), ×400 (**c**, **f**, **g**)

After 48 h of incubation with the mycobacteria (Fig. 2), we observed damage in the histologic structure: inflammatory infiltrates composed of histiocytes and aggregates of foamy macrophages, as well as nuclear fragmentation of the PMN cells (“nuclear dust”). Rupture of the alveolar septa, vascular congestion and thickening of the alveolar septa in other areas were also noticed (Fig. 2a–g). Some of the foamy macrophages presented nuclear changes, such as pyknosis, karyolysis and karyorrhexis (Fig. 2). Abundant mycobacteria were observed in the alveolar septum infecting the type II pneumocytes and in the areas of confluence of foamy macrophages (Fig. 2). Langhans multinucleated cells were also observed (Fig. 2e, g); mycobacterial fragments inside the multinucleated giant cells were sometimes observed.

**Fig. 3:**
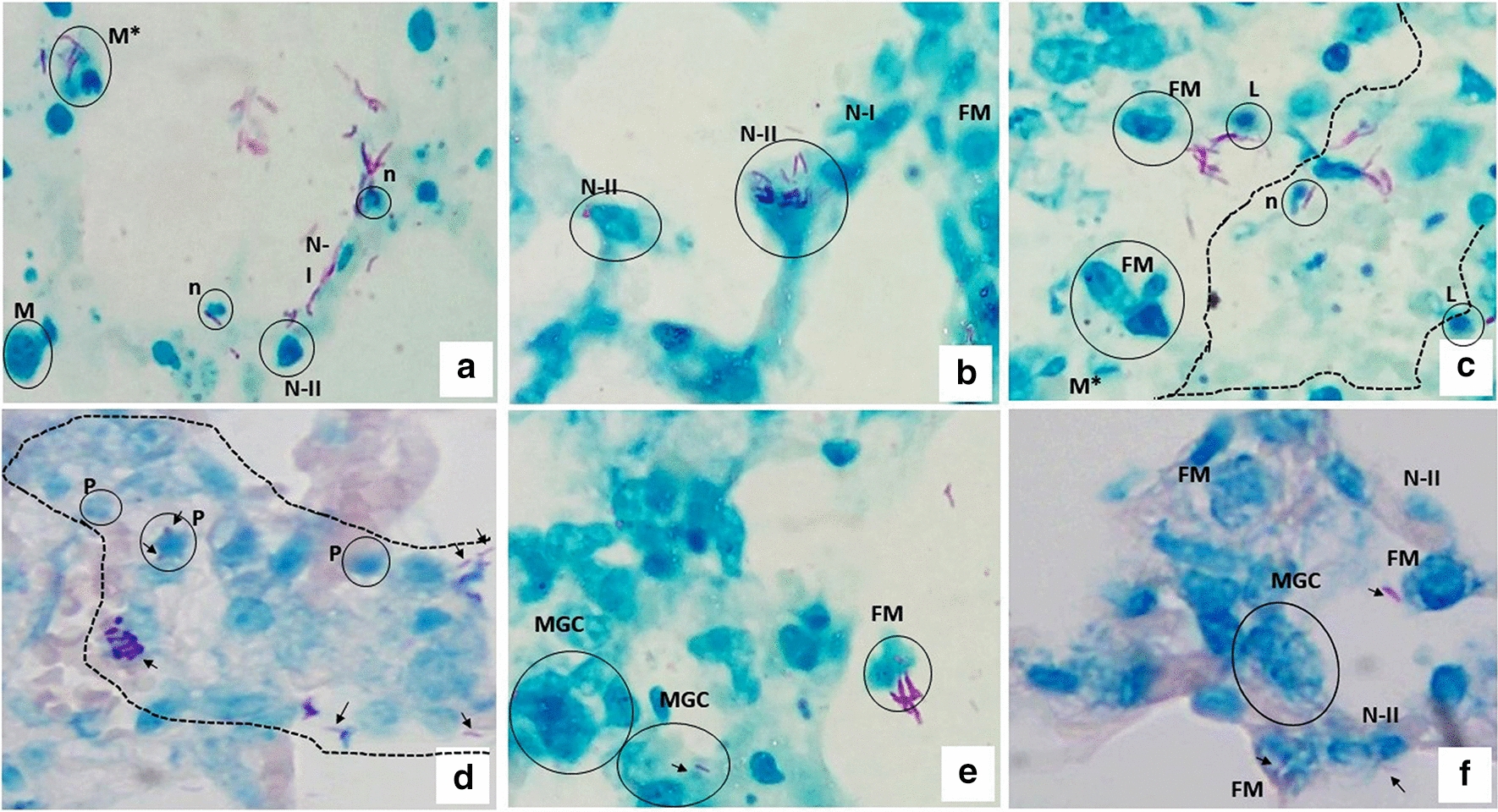
Interactions of *M. abscessus* with epithelial cells in the infected PCLS. **a**–**c** *M. abscessus*, stained in pink, can be found isolated or forming aggregates in the alveolar space; mycobacteria can be observed interacting directly with type I pneumocytes (NI), type II pneumocytes (N-II), neutrophils (N), lymphocytes (L), and foamy macrophages (FM), some of which demonstrate karyorrhexis (M*); in addition, cell detritus with nuclear fragments are observed (dotted area in **c**). **d** A cluster of foamy macrophages (dotted area), some of which show nuclear pyknosis (P); mycobacterial fragments are identified with arrows. **e**, **f** Multinucleated giant cell (MGC) interacting with mycobacteria (arrows). ZN staining (×40). Total magnification

**Fig. 4:**
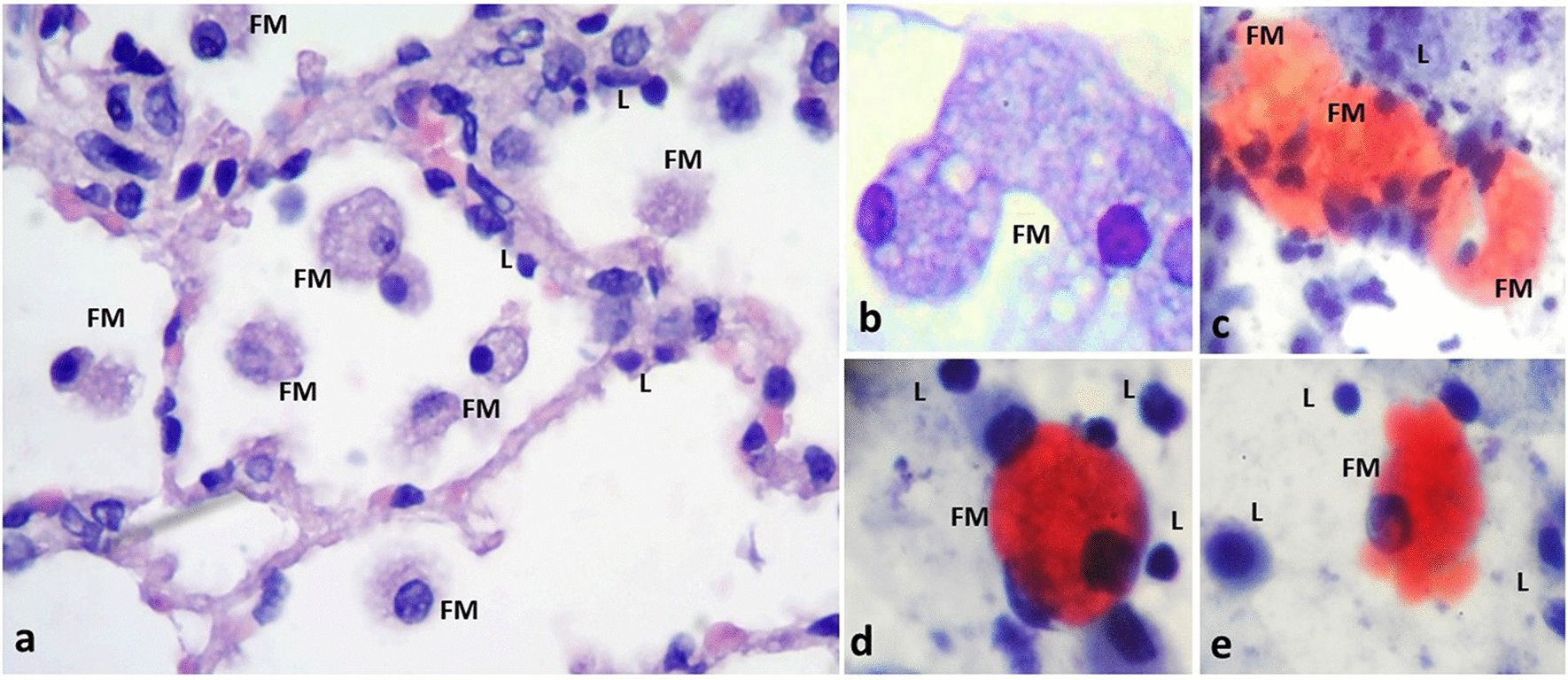
Presence of foamy macrophages in the infected PCLS. Inflammatory infiltrates with aggregates of foamy macrophages were frequently observed in the PCLS infected with *M. abscessus*. **a** Alveolar spaces with abundant foamy macrophages (FM). **b** Cellular detail of FM containing multiple intracytoplasmic vacuoles. Foamy macrophages (FM) can be found in aggregates (**c**), or isolated (**d**, **e**), with positive staining for accumulation of lipids in their cytoplasm; lipid-laden cells are a distinctive hallmark of mycobacterial infections. Mononuclear cells, predominantly lymphocyes (L) are also observed. H&E staining (**a**, **b**). Oil red-O staining (**c**–**e**). Total magnification: ×10 (**a**), ×40 (**b**–**e**)

The interactions of *M. abscessus* with inflammatory cells and pneumocytes in the alveolar septa are shown in Fig. 3a–f. *M. abscessus* interacts directly with type I and type II pneumocytes, neutrophils, lymphocytes, and macrophages. Bacilli were observed isolated in the alveoli and alveolar space but also with a tendency to form aggregates in the alveolar septum, near to or in close contact with the epithelial cells; they were also found between cellular debris in areas of inflammation and in close contact with macrophages. We observed the infection of macrophages and type I pneumocytes, as bacilli were seen inside of these cells. Foamy macrophages filled with lipid-containing bodies were frequently found in frozen sections from infected PCLS stained with Oil red-O (Fig. 4).

**Fig. 5:**
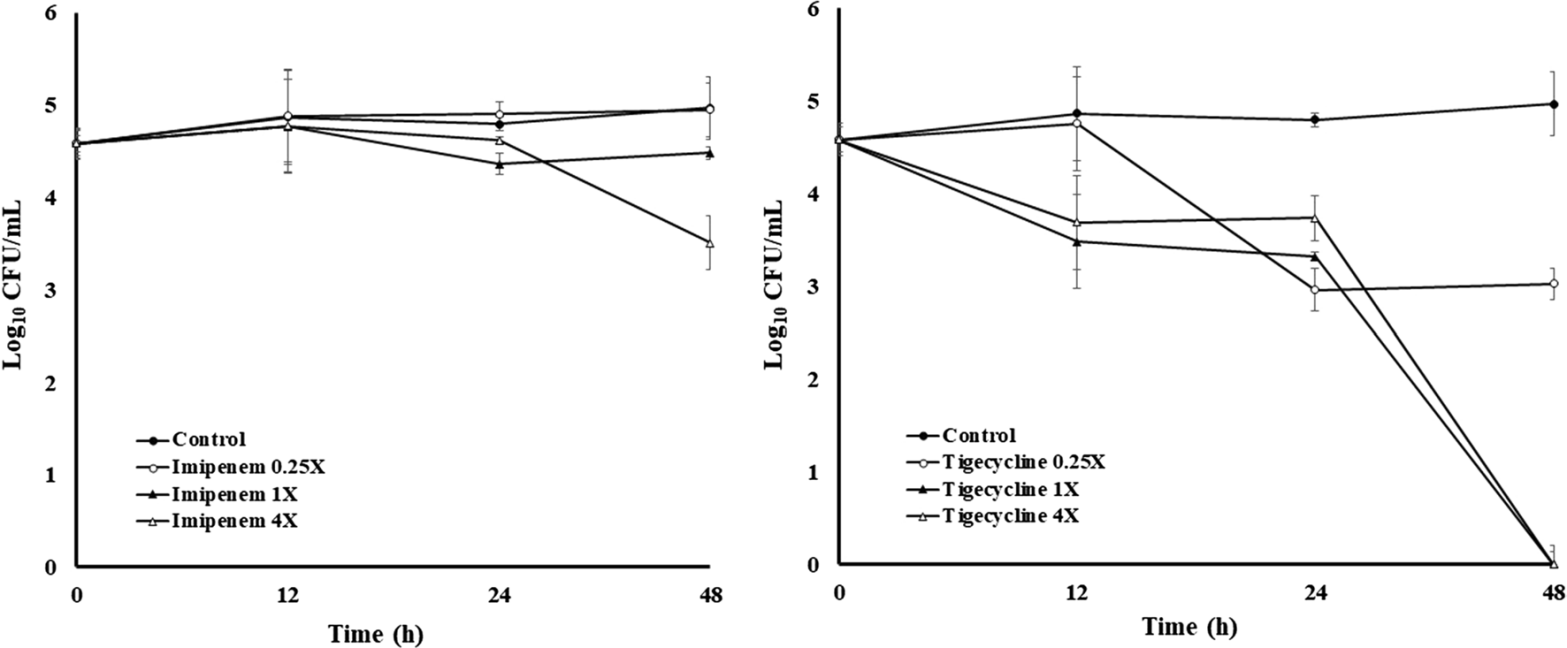
Intracellular activity of antimicrobials in *M. abscessus*-infected murine lung slices. Bacterial counts were performed at 12, 24, and 48 h after infection. Error bars represents the mean of three measurements

To confirm the usefulness of this model, we studied the intracellular effect of imipenem and tigecycline on the infected PCLS. The MIC values for tigecycline and imipenem were 1 and 16 μg/mL, respectively, for this *M. abscessus* strain. Bactericidal activity and bacteriostatic activity were defined as ≥ 3log_10_ and < 3log_10_ reduction of total count of CFU/mL, respectively, in comparison with the initial inoculum after 12, 24, 36 and 48 h of incubation according to standard guides [31]. Bacteriostatic intracellular activity on the infected slices was observed with imipenem. As shown in Fig. 5, the intracellular CFU count decreased only 1 log at 48 h at the highest drug concentration used. A Student’s t-test showed no significant difference between the imipenem-treated slices and the nontreated control.

A better result was obtained with tigecycline, which showed a dose-and time-dependent activity and a bactericidal effect at 48 h that reached a reduction of > 4log_10_ CFU/mL (Fig. 5). A post hoc test showed a statistically significant difference between the control and those slices treated with tigecycline at 1× and 4× the MIC (P < 0.05).

**Fig. 6:**
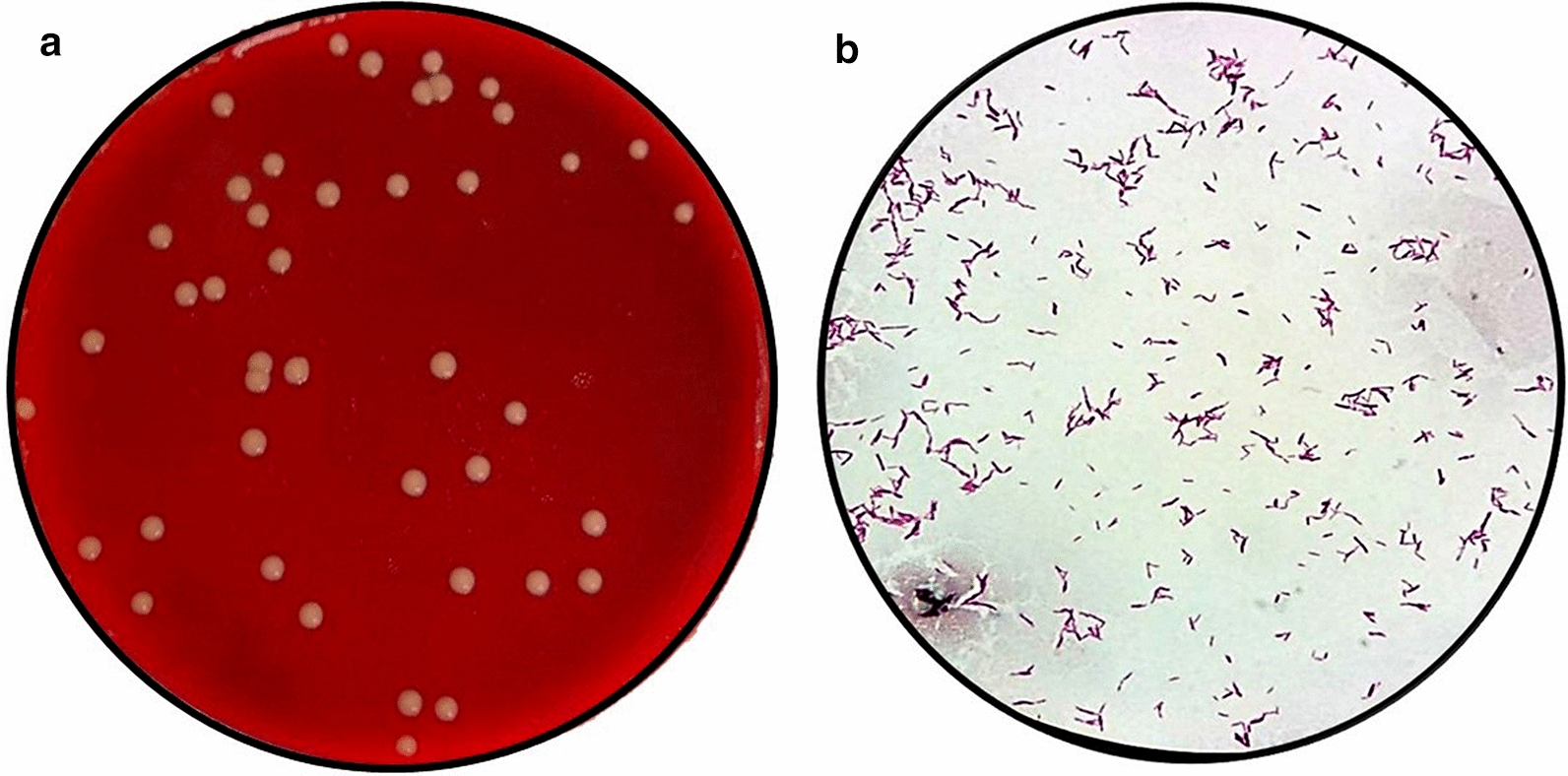
Culture of the lysates from PCLS infected with *M. abscessus*. **a** Typical colony morphology of mycobacteria from the culture of the lysate of murine lung slices infected with *M. abscessus*. **b** Microscopic morphology of acid-resistant acid bacilli from the same bacterial colonies (arrowheads). ZN staining (×100)

We also corroborated that intracellular bacteria recovered from the infected PCLS at equivalent time points were viable *M. abscessus* by lysing and plating the tissue homogenate in blood agar, as described in “Materials and methods” section. After incubation, the growth of round bacterial colonies with the typical morphology of mycobacteria was observed (Fig. 6a). The subsequent ZN staining of the smears from these colonies showed the presence of the mycobacterial bacilli (Fig. 6b). These results and the previously described morphological findings support the utility of the experimental infection model.

## Discussion

Due to the antimicrobial resistance shown by *M. abscessus*, it is necessary to study and analyze new antibacterial candidates using physiologically relevant models. Here, we evaluated a preclinical model for *M. abscessus* infection using PCLS and validated it by determining the antimicrobial effects of imipenem and tigecycline. A stable and sustained growth of the bacteria was observed inside the tissue up to 48 h post-infection, allowing us to assess the antimicrobial activity of these drugs.

Other approaches to study the intracellular activity of drugs against *M. abscessus* have been performed in vitro using a variety of macrophages, including bone marrow-derived macrophage, J774 and THP-1 cells [32–34].

Animal models have also been used to evaluate the antimycobacterial efficacy of drugs against *M. abscessus* infection; however, only severely immunocompromised strains of mice such as GKO or SCID have shown acceptable levels of infection [35–37]. The advantage of these mouse models is that *M. abscessus* progressively develops a high level of infection, allowing the detection of significant differences between the *M. abscessus* control and the drug-treated groups [35].

Nevertheless, in vivo studies have sometimes led to inconsistent results [10]; for instance, bedaquiline treatment reduced the CFUs by 2 logs in a SCID mouse model but was almost inactive in nude mice [35, 37]. However, the main limitations of animal models in the search for antimicrobial activity against NTM are the large number of experimental animals regularly used, and the high costs of housing and handling the genetically modified mice [35, 38, 39].

Additionally, it has been reported that it takes up to 60 days for the study of the infection using the C57BL/6 and GKO mice [36]. These facts contrast sharply with the number of animals used in the present work and the time taken; here we used only ten mice, including those used for standardization, and the ex vivo time course of infection induced by *M. abscessus* was only a few days. In contrast, we were able to observe the histopathological characteristics of *M. abscessus*-induced damage and the pathogen’s interactions with lung parenchyma cells after a short time post-infection (6–48 h), while animal models need 10 to 60 days to develop lung damage [35, 36, 38, 40]. Studies with *M. tuberculosis* [19, 20] and other pathogens [41–45] using PCLS or tissue explants, where tissue lesions or inflammatory infiltrates are observed at 24–48 h post-infection, support the advantage of these ex vivo models.

Findings such as the presence of foamy macrophages or nuclear fragmentation of neutrophils seen before 24 h in our model corroborate those results reported by Bernut et al. [46] who described the same results using zebrafish embryos.

At 48 h post-infection, we observed the presence of Langhans cells, which have not been previously reported in murine models of *M. abscessus* infection. These multinucleated giant cells are a histopathological landmark for mycobacterial infections, where they seem to have an important role in restricting mycobacterial growth [47, 48], although they are not specific, as they are also present in numerous granulomatous diseases [49], including those caused by nontuberculous mycobacteria [50, 51]. Langhans cells have been reported in granulomatous infiltrates in *M. abscessus*-induced cutaneous and pulmonary infections in immunocompromised patients [52–55].

At the histopathological level, in one of the most extensive studies where nine different transgenic murine models were used to analyze the ability of *M. abscessus* to induce infection, granuloma formation was reported, but multinucleated giant cells were not seen [35]. In contrast, granulomas with Langhans multinucleated cells were found in BALB/c infected with the vaccine strain of *M. bovis* (the Bacillus Calmette-Guérin vaccine, BCG-1). However, the granulomas containing these multinucleated Langhans cells were obtained from splenic tissues but not from the lungs of the infected mice [56]. In general terms, the histopathological findings that we describe here are akin to those reported by other investigators [35, 36, 40, 57].

Ordway et al. [36] infected knockout mice with a high-dose aerosol of* M. abscessus*, and demonstrated peribronchiolar inflammatory infiltrates at 15 days; at day 30, they observed granulomas composed of aggregates of lymphocytes and foamy cells; and by day 60, the granulomas were larger, as well as the inflammatory infiltrates. When guinea pigs were infected in the same way, a more severe granulomatous inflammation in the lungs was observed at day 60, and it was characterized by sheets of epithelioid macrophages and organized aggregates of lymphocytes that infiltrated septal walls and filled alveolar spaces.

De Groote et al. [57] developed an animal model of *M. abscessus* chronic infection using granulocyte–macrophage colony-stimulating factor knockout (GM-CSF KO) mice. They reported inflammation of the bronchioles and alveolar architecture alterations in infected animals, with presence of macrophages and neutrophils. Acid-fast bacilli were observed inside macrophages, as well as some free bacilli in alveoli too, and after 4-months of chronic infection, large accumulations of foamy macrophages within the alveoli were observed.

In our PCLS model, the uninfected slices kept their tissue architecture intact, while the histological analysis of PCLS infected with *M. abscessus* showed the presence of polymorphonuclear cells, epithelioid cells, foamy macrophages, multinucleated giant cells, and the signs of an early granulomatous inflammation after 6 and 12 h post-infection. We also observed acid-fast bacilli within macrophages, and isolated bacillus or forming aggregates in the alveolar spaces. The infected PCLS showed tissue destruction, causing the loss of the histological architecture, rupture of the alveolar septa, and areas of inflammatory aggregates with nuclear PMN fragmentation, as well as vascular congestion and thickening of the alveolar septa at 48 h post infection. The histological changes found in the in vivo models are similar to those found in the BALB/c mice PCLS model infected with *M. abscessus*, but at shorter infection time and it indicates that our model responds in a very similar way as the lung tissue does in an animal model.

An additional advantage of the ex vivo infection model using PCLS is that we used normal BALB/c mice, while murine infection models to study *M. abscessus* require expensive genetically modified mice, thus our methodology decreases the cost per experiment.

The development of our ex vivo 3D model enables the study of an *M. abscessus* experimental infection within a physiological milieu and provides the opportunity to study the infection process not only in lung tissue from laboratory animals but also in human lung tissue, as reported by Ganbat et al. [19] who used a similar ex vivo tissue culture model for mycobacterial infections. These authors infected the human lung tissues samples with two strains of each three different mycobacterial species, including *M. abscessus*, and focused on the very early onset of TB infection, and on the specific interactions between mycobacteria and the cells of the lung. The morphologic comparisons between freshly obtained and cultured ex vivo lung specimens showed no noticeable differences, like our results. The authors found, as we also did, that mycobacteria can infect different cell types, including macrophages, neutrophils, monocytes, and type II pneumocytes.

The presence of foamy macrophages in PCLS infected with *M. abscessus* was clearly demonstrated (Fig. 4). Foamy macrophages are a distinctive characteristic of granulomas associated with virulent mycobacteria; these cells have been observed in different inflammatory conditions, including infectious and noninfectious diseases, such as natural and experimental TB in particular [58]. The formation of foamy macrophages has been described in models of infection with *M. abscessus* both, in vitro [59, 60], and in vivo [61, 62]. It has been suggested that the induction of foamy macrophages results in an intracellular microenvironment that allows the persistence of mycobacteria, and that the fatty acids that accumulated in their cytoplasmic vacuoles represent a source of nutrients for the bacilli [63].

Our findings on the ability of *M. abscessus* to infect lung cells in PCLS allowed us to evaluate the effects of two well-known antibacterial drugs. Imipenem, an antibiotic that is part of the multidrug therapy recommended for infections by *M. abscessus*, showed little effect against bacteria at a low MIC concentration, but a bacteriostatic effect at medium and high concentrations. This was an expected result, as other studies have shown the same behavior when intracellular activity was investigated [32, 64]. We also evaluated tigecycline, which has previously demonstrated good in vitro MICs against *M. abscessus* isolates and has been used as a rescue treatment for *M. abscessus* and *Mycobacterium chelonae* complicated infections [8]. In this study, tigecycline was bactericidal after 24 and 48 h post-infection at 1× and 4× the MIC value (P < 0.05). Previously, this compound had already been demonstrated to have an observable bactericidal effect against resistant *M. abscessus* in a hollow-fiber model system for pulmonary disease [65], as well as intracellular activity in a THP-1 macrophage model [32].

## Conclusions

In conclusion, PCLS represent a useful 3D model for the study of ex vivo infection with *M. abscessus* and the activity of new antimycobacterial drugs. This model has the simplicity and reproducibility of in vitro models, but its major advantage is the presence of more than forty differentiated cell types with metabolic capability, polarization, and extracellular elements found in in vivo models. Furthermore, this model complies with the 3R’s Principle [66, 67] and provides the opportunity for testing new drugs against *M. abscessus*, decreasing the use of costly and tedious animal models.

## Data Availability

The datasets used and/or analyzed during the current study are available from the corresponding author on reasonable request.

## Abbreviations

TB: Tuberculosis
PCLS: Precision cut lung slices
NTM: Nontuberculous mycobacteria
CFU/mL: Colony forming units/mL
MIC: Minimal inhibitory concentration
CLSI: Clinical and Laboratory Standards Institute
ZN: Ziehl–Neelsen
H&E: Hematoxylin and eosin
